# Relationship Between Health Literacy and Social Support and the Quality of Life in Patients With Cancer: Questionnaire Study

**DOI:** 10.2196/17163

**Published:** 2020-03-19

**Authors:** Rei Kobayashi, Masato Ishizaki

**Affiliations:** 1 Interfaculty Initiative in Information Studies The University of Tokyo Tokyo Japan

**Keywords:** health literacy, social support, quality of life, neoplasms, health communication

## Abstract

**Background:**

Low health literacy is associated with factors such as not taking medication as prescribed as well as poor health status and increased hospitalization and mortality risk, and has been identified as a risk factor for decreased physical function in older individuals. Health literacy is becoming an increasingly important issue because of the increased number of people affected by cancer who must make complicated treatment decisions. Health literacy has been shown to be positively associated with quality of life (QOL), and social support has been identified as important for addressing health-related problems and reducing the relative risk of mortality in patients with cancer. However, few studies have examined the relationship between health literacy, social support, age, and QOL.

**Objective:**

The aim of this study is to examine the effects of health literacy, social support, and age on the QOL of patients with cancer.

**Methods:**

An anonymous, self-administered online questionnaire was conducted from March 28 to 30, 2017, in Japan on patients with lung, stomach, or colon cancer that were voluntarily registered with an internet survey company. The survey covered basic attributes, health literacy, social support, and QOL. The European Health Literacy Survey Questionnaire, a comprehensive measure of health literacy instrument, was used to measure health literacy; the Japanese version of the Social Support Scale was used to measure social support; and the Japanese version of the Functional Assessment of Cancer Therapy-General (7-item version) assessment tool was used to measure QOL.

**Results:**

A total of 735 survey invitations were randomly sent to patients with lung, stomach, or colorectal cancer, and responses were obtained from 619 (82.2% response rate). Significant effects on the QOL in patients with lung, stomach, or colon cancer were observed for health literacy, social support, and age, and for the interactions of health literacy and social support and of social support and age. Health literacy, social support, and the interaction between these variables also showed a significant effect on the QOL in patients 50 years or older, but not on those younger than 50 years.

**Conclusions:**

The results of this study revealed that higher health literacy, social support, and age were associated with the QOL in patients with cancer. In addition, the relationship with QOL was stronger for social support than for health literacy. These findings suggest the importance of health literacy and social support and indicate that social support has a greater effect on QOL than does health literacy, while the QOL in patients with cancer aged younger than 50 years was lower than that of those 50 years or older. Therefore, elucidating the needs of these patients and strengthening social support based on those needs may improve their QOL.

## Introduction

### Health Literacy and Quality of Life in Patients With Cancer

Health literacy is the ability to obtain, understand, evaluate, and use information about health and medical care [1]. Low health literacy is associated with poor health status, limited access to health care, increased use of expensive health care services, and high mortality rates [2-4]. In addition, it is associated with not taking medication as prescribed [5,6] as well as increased hospitalization and mortality risk in patients with a history of heart failure [7]. Among older individuals, low health literacy has been identified as a risk factor for decreased physical function [8]. Moreover, in terms of prevention, low health literacy has been found to be associated with low participation in colorectal cancer screening [9].

Increasing numbers of people worldwide are afflicted by and die from cancer [10-13]. Patients with cancer must make complicated decisions that have a major effect on their treatment and future; thus, health literacy is a particularly important issue [14]. Low health literacy can result in the misunderstanding of a disease, inadequate treatment due to the inability to communicate satisfactorily with health care personnel, and an inability to comply with treatment plans [15-17].

The World Health Organization defines quality of life (QOL) as an individual’s perception of their position in life in the context of the culture and value systems where they live and in relation to their goals, expectations, standards, and concerns [18]. Considering the continuous care required for patients with cancer, including the use of medical care services, routine health management, and end-of-life medical care, health literacy has a major effect on QOL [19-22]. In a previous study [23], health literacy was found to be positively associated with QOL.

Efforts to increase health literacy appear in national policies in the form of programs such as the National Action Plan to Improve Health Literacy in the United States [24] and The Japan Vision: Health Care 2035 [25]. However, the results have been less than satisfactory [26]. Moreover, health literacy does not increase with health care experience [27]. The findings of these previous reports suggest that improving health literacy is not a straightforward task.

### Social Support and Quality of Life in Patients With Cancer

The QOL in patients with cancer has been found to be affected by the attributes and psychological and physical health of the individual [28-30], and by symptoms and levels of high anxiety after cancer treatment [31]. These factors have been found to be important in facilitating adaptation to daily life following treatment [32]. In addition, social support has been identified as an important means of addressing health-related problems [33]. A study of patients with lung cancer suggested that social and emotional support are important for QOL [34]. Moreover, strong social support has been shown to reduce the relative risk of mortality in patients with cancer [35]. A study of aged survivors of cancer found that QOL was high in individuals with strong emotional support and low in those with weak emotional support [36]. Further, in a study of aged patients with cancer undergoing chemotherapy, QOL was higher in those with strong social support [37], and in patients with colon or rectal cancer, QOL increased with stronger social support [38]. However, to our knowledge, health literacy and QOL and social support and QOL have only been studied separately; few studies have examined the relationship between health literacy and social support.

### Health Literacy, Social Support, Age, and Quality of Life in Patients With Cancer

Lee (2004) [33] posed a research question on health literacy, social support, and QOL regarding whether social support can mitigate low health literacy and improve QOL. In addition, a similar study found that low health literacy and a high degree of social isolation were independently associated with increased mortality risk [39]. A high degree of social isolation and weak social support may overlap, and mortality can be understood as an objective number. The present study takes Lee’s (2004) [33] research question seriously, and in doing so, attempts to clarify the relationship between QOL and health literacy and social support in patients with cancer (research question 1).

Lee (2009) [40] examined health literacy, social support, and QOL in relation to Medicare in the United States, and found positive correlations between high health literacy, high social support, and better QOL. An examination of cancer mortality risk according to age reported that the risk of cancer mortality increases with age. According to the American Cancer Society, 80% or more of patients diagnosed with cancer in the United States are 55 years or older [41]. In the United Kingdom, the incidence of cancer rapidly increases beginning at around 55 years of age, according to Cancer Research UK [42]. In Japan, the risk of cancer for both men and women starts to increase when people are in their 50s [43]. Consequently, in addition to research question 1, this study examined the relationship between QOL and health literacy and social support in patients aged 50 years or older and patients younger than 50 years, and whether there were differences between these two groups (research question 2). This approach was based on facts that Smith et al (2018) [39] previously looked at in patients 50 years or older, and cancer risk begins to increase when people reach their 50s.

## Methods

### Participants

An anonymous, self-administered, online questionnaire survey was conducted from March 28 to 30, 2017, by an internet survey company in Japan. The survey participants were recruited from voluntarily registered patients with lung, stomach, or colon cancer. These types of cancer were selected because they ranked as the top three for cancer mortality in Japan [43]. In total, 735 potential respondents (diagnosed with lung, stomach, or colon cancer, and between the ages of 20-69 years) were randomly invited by email to participate in an anonymous, cross-sectional online survey, and 619 accepted the invitation and responded (collection rate 82.2%). The survey data were anonymized and managed so that individuals involved in the study could not be identified. This study was approved by the institutional review board of the University of Tokyo Interfaculty Initiative in Information Studies.

### Measures

The survey covered basic attributes, health literacy, social support, and QOL. The instrument used to measure health literacy was the European Health Literacy Survey Questionnaire (HLS-EU-Q47), a comprehensive measure of health literacy. The reliability and validity of the questionnaire has been confirmed for not only the original version, but also the Japanese translation (HLS-EU-Q47 Japanese Version) [44]. The HLS-EU-Q47 is not targeted at patients with cancer, and its items can be divided into three areas: health care, disease prevention, and health promotion. Because the participants in this study were patients with cancer, the survey was narrowed to 16 health care items related to the health care experiences of patients with cancer. The items asked whether the participants were able to find information on their disease and its treatment, whether they understood the information they obtained from physicians, and whether they understood the medications they received.

To measure social support, the Japanese version of the Social Support Scale [45,46] was used. The reliability and validity of the Japanese version of the scale have been validated. The Japanese version of the Functional Assessment of Cancer Therapy-General (7-item version) (FACT-G7) version 4 assessment tool was used to measure QOL [47]. The Functional Assessment of Cancer Therapy-General (FACT-G) has been used in studies of QOL and health literacy in patients with cancer [23]. Although the FACT-G7 is an abbreviated version of the established FACT-G, its effectiveness has been confirmed to be interchangeable with that of the FACT-G [48]. The FACT-G7 was used in this study to minimize the burden on the survey participants.

### Analysis

For health literacy, responses to the 16-item Japanese version of the HLS-EU-Q47 questionnaire were obtained as a score of 1 to 4 points on a 4-point Likert scale (from very easy to very difficult, reverse-scored items) or 0 (do not know). For social support, responses to the 12-item Japanese version of the Social Support Scale were obtained as a score of 1 to 5 points on a 5-point Likert scale (from disagree to agree). Responses to the 7-item Japanese version of the FACT-G7 (version 4) were obtained as a score of 1 to 5 points on a 5-point Likert scale (from very much to not at all). Reverse-scored items were adjusted so that 5 indicated a high QOL and 1 indicated a low QOL. The mean score for the seven items was used for QOL. All the variables of each construct were calculated as the sums of the item scores.

A multiple regression analysis performed with QOL as the dependent variable used age, sex, household income, educational level, time since diagnosis, type of cancer, and disease stage as dummy variables when the survey was conducted, and the standardized values (z scores) of the total score for the 16 health literacy items and the total score for the 12 social support items were used as independent variables. Statistical analysis was performed using SPSS version 24 (IBM Corp, Armonk, NY) and R 3.6.1 software (R Foundation for Statistical Computing, Vienna, Austria).

## Results

### Participants

The characteristics of the survey participants are shown in Table 1. The mean health literacy, social support, and QOL values were 2.25 (SD 0.71), 3.52 (SD 0.94), and 3.41 (SD 0.80), respectively. Dividing the participants into two groups based on age, the values were 2.29 (SD 0.67), 3.63 (SD 0.92), and 3.66 (SD 0.78), respectively, for those aged 50 years or older and 2.19 (SD 0.78), 3.35 (SD 0.95), and 3.05 (SD 0.69), respectively, for those younger than 50 years.

**Table 1:** Characteristics of the survey participants.

Characteristics			Total (N=619), n (%)		Age ≥50 years (n=376), n (%)		Age <50 years (n=243), n (%)
**Sex**							
	Male	477 (77.1)		317 (84.3)		160 (65.8)	
	Female	142 (22.9)		59 (15.7)		83 (34.2)	
**Household income (million JPY)**							
	<4	157 (25.4)		107 (28.5)		50 (20.6)	
	≥4 and <8	209 (33.8)		123 (32.7)		86 (35.4)	
	≥8	178 (28.8)		110 (29.3)		68 (28.0)	
	Unknown or not available	75 (12.1)		36 (9.6)		39 (16.0)	
**Educational attainment**							
	Less than university degree	253 (40.9)		159 (42.3)		94 (38.7)	
	University/graduate degree	354 (57.2)		208 (55.3)		146 (60.1)	
	Other or unknown	12 (1.9)		9 (2.4)		3 (1.2)	
**Time since diagnosis**							
	<6 months	99 (16)		51 (13.6)		48 (19.8)	
	≥6 months and <1 year	82 (13.2)		42 (11.2)		40 (16.5)	
	≥1 year and <2 years	182 (29.4)		113 (30.1)		69 (28.4)	
	≥2 years	256 (41.4)		170 (45.2)		86 (35.4)	
**Site of primary tumor**							
	Lung	119 (19.2)		57 (15.2)		62 (25.5)	
	Stomach	206 (33.3)		124 (33.0)		82 (33.7)	
	Colon	294 (47.5)		195 (51.9)		99 (40.7)	
**Cancer stage**							
	I	250 (40.4)		151 (40.2)		99 (40.7)	
	II	133 (21.5)		65 (17.3)		68 (28.0)	
	III	103 (16.6)		64 (17.0)		39 (16.0)	
	IV	50 (8.1)		32 (8.5)		18 (7.4)	
	Unknown	83 (13.4)		64 (17.0)		19 (7.8)	

### Quality of Life in Patients With Cancer

A multiple regression analysis including dummy variables was performed with QOL as the dependent variable, and the model shown in Table 2 was obtained (*F*_22,596_=811.99; *P*<.001; adjusted R^2^=0.281). In this model, the standard partial regression coefficients were significant for age, health literacy, social support, interaction of health literacy and social support, and interaction of social support and age.

**Table 2:** Multiple regression analysis of quality of life for all participants.

Variables	B	SE	Beta	*P* value	Variance inflation factor
(Constants)	3.453	0.110	N/A^a^	<.001	N/A
Female (reference male)	0.105	0.070	.055	.13	1.140
Age ≥50 years (reference <50 years)	0.526	0.061	.320	<.001	1.184
Household income ≥4 and <8 million JPY (reference <4 million JPY)	–0.040	0.074	–.024	.59	1.637
Household income ≥8 million JPY (reference <4 million JPY)	–0.026	0.079	–.015	.79	1.704
Household income unknown or not available (reference <4 million JPY)	–0.187	0.098	–.076	.06	1.373
University/graduate degree (reference less than university degree)	0.035	0.060	.022	.55	1.158
Other/unknown (reference less than university degree)	0.112	0.208	.019	.59	1.101
Time since diagnosis ≥6 months and <1 year (reference <6 months)	–0.111	0.104	–.047	.29	1.657
Time since diagnosis ≥1 and <2 years (reference <6 months)	0.086	0.087	.049	.32	2.100
Time since diagnosis ≥2 years (reference <6 months)	–0.006	0.083	–.003	.95	2.244
Stomach cancer (reference lung cancer)	0.016	0.081	.010	.84	1.940
Colon cancer (reference lung cancer)	0.199	0.078	.124	.01	2.019
Stage II (reference Stage I)	–0.301	0.075	–.154	<.001	1.266
Stage III (reference Stage I)	–0.237	0.082	–.110	.004	1.242
Stage IV (reference Stage I)	–0.622	0.109	–.211	<.001	1.167
Stage unknown (reference Stage I)	–0.020	0.090	–.008	.82	1.254
Health literacy	0.106	0.029	.132	<.001	1.106
Social support	0.169	0.029	.210	<.001	1.096
Health literacy * Social support	–0.050	0.025	–.073	.047	1.139
Health literacy * Age	0.069	0.057	.043	.23	1.084
Social support * Age	0.196	0.057	.119	.001	1.039
Health literacy * Social support * Age	–0.082	0.048	–.061	.09	1.126

^a^N/A: not applicable.

### Quality of Life According to Age

A multiple regression analysis including dummy variables was performed for individuals aged 50 years or older with QOL as the dependent variable, and the model shown in Table 3 was obtained (F_18,357_=7.33; *P*<.001; adjusted R^2^=0.233).

In this model, the standard partial regression coefficients were significant for health literacy, social support, and the interaction between health literacy and social support.

**Figure 1:**
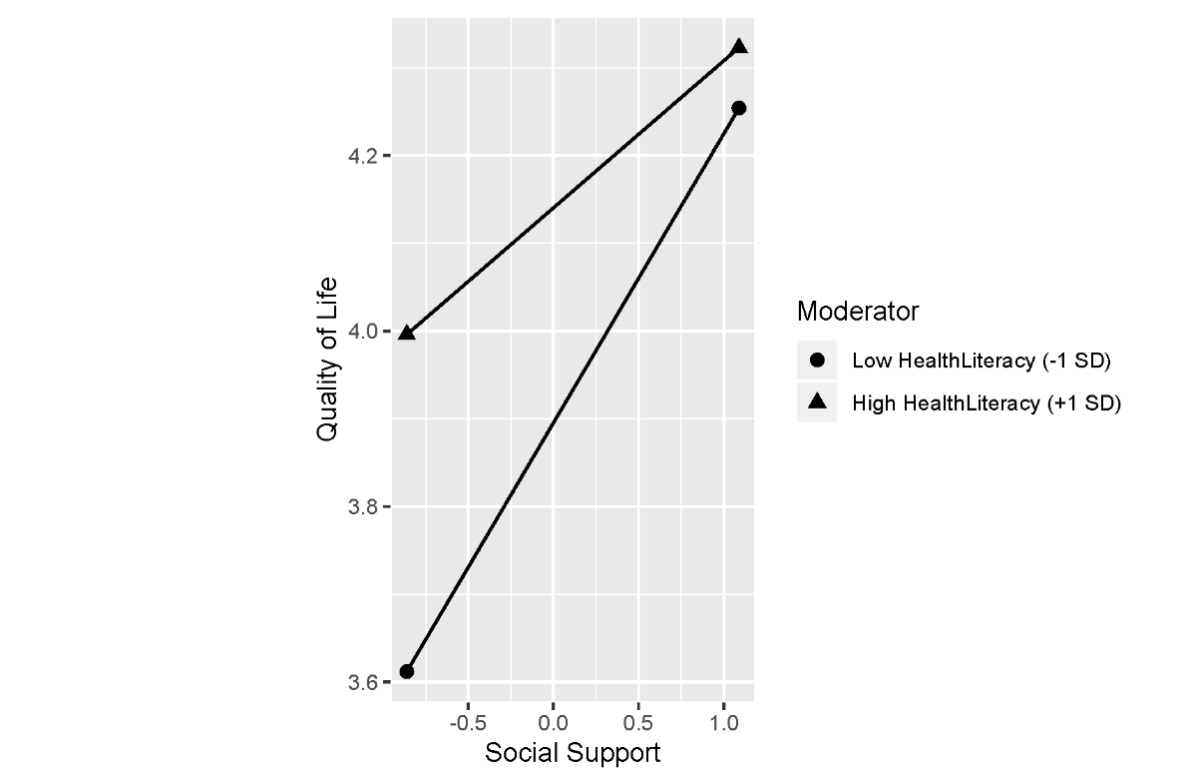
Relationship between health literacy and social support and the quality of life in patients 50 years or older.

The results of a simple slope analysis for the interaction of health literacy and social support are shown in Table 4 and Figure 1. For health literacy and social support, a value below the mean was considered low, and a value equal to or above the mean was considered high.

A significant association was seen between QOL and social support, regardless of the level of health literacy (*P*<.001). However, the coefficient for social support was larger when health literacy was low compared with when it was high.

Next, a multiple regression analysis including dummy variables was performed with QOL as the dependent variable for participants younger than 50 years; the results were not significant (F_18,224_=1.63, *P*=.06).

**Table 3:** Multiple regression analysis of quality of life in patients aged 50 years or older.

Variables	B	SE	Beta	*P* value	Variance inflation factor
(Constant)	3.647	0.150	N/A^a^	<.001	N/A
Female (reference male)	0.094	0.103	.044	.36	1.115
Household income ≥4 and <8 million JPY (reference <4 million JPY)	–0.110	0.094	–.066	.24	1.541
Household income ≥8 million JPY (reference <4 million JPY)	–0.059	0.099	–.034	.56	1.617
Household income unknown or not available (reference <4 million JPY)	–0.388	0.135	–.146	.004	1.255
University/graduate degree (reference less than university degree)	0.059	0.078	.037	.45	1.204
Other or unknown education (reference less than university degree)	0.068	0.245	.013	.78	1.120
Time since diagnosis ≥6 months and <1 year (reference <6 months)	–0.071	0.148	–.028	.63	1.737
Time since diagnosis ≥1 and <2 years (reference <6 months)	0.093	0.118	.055	.43	2.354
Time since diagnosis ≥2 years (reference <6 months)	0.067	0.113	.043	.55	2.522
Stomach cancer (reference lung cancer)	0.089	0.113	.053	.43	2.256
Colon cancer (reference lung cancer)	0.276	0.108	.176	.01	2.324
Stage II (reference Stage I)	–0.431	0.105	–.208	<.001	1.254
Stage III (reference Stage I)	–0.251	0.107	–.121	.02	1.279
Stage IV (reference Stage I)	–0.697	0.138	–.249	<.001	1.180
Stage unknown (reference Stage I)	–0.121	0.107	–.058	.26	1.294
Health literacy	0.125	0.039	.150	.002	1.087
Social support	0.246	0.038	.306	<.001	1.093
Health literacy * Social support	–0.083	0.036	–.109	.02	1.085

^a^N/A: not applicable.

**Table 4:** Simple slope analysis for interaction of health literacy and social support.

Variables	Simple slope	SE	*t*_357_ (2-tailed)	*P* value
Low health literacy (–1 SD)	0.33	0.05	6.56	<.001
High health literacy (+1 SD)	0.17	0.05	3.34	<.001

## Discussion

### Relationship Between Health Literacy and Social Support and the Quality of Life in Patients With Cancer

We identified significant effects of health literacy, social support, age, interaction of health literacy and social support, and interaction of social support and age on QOL for the patients with lung, stomach, or colon cancer. We also found that the effect of social support and age is stronger than that of health literacy when controlling for the effects of other independent variables. In addition, health literacy, social support, and their interaction were found to have a significant effect on QOL. Moreover, the effect of social support is stronger for low health literacy than for high health literacy in those 50 years or older, while there is no effect on the QOL in those younger than 50 years.

Studies involving patients with cancer have indicated that health literacy with respect to treatment is important for understanding a disease [14-16], and that health literacy is positively associated with QOL [23]. Previous results on social support have indicated that among older patients with cancer undergoing chemotherapy, QOL was higher for those with strong social support [37]. In this study, the levels of health literacy and social support were related to QOL. Moreover, the association was stronger for social support than for health literacy. These findings suggest that both health literacy and social support are important considerations for the QOL in patients with cancer.

Health literacy can be considered a patient resource [49]. Although efforts have been undertaken to increase health literacy, this is not easily accomplished [24-26]. Social support, on the other hand, is a resource provided by the people with whom the patient associates. Support from family members, friends, and acquaintances is what the patient is most familiar with, and thus, it is important to continue to strengthen such support. However, this support varies depending on the individual, and those providing support may have their own physical, emotional, social, or financial problems [50]. Consequently, a need for consulting services that specialize in social support has been suggested [51].

Efforts to provide a system of social support not dependent on individual circumstances have been implemented. Maggie’s [52] centers were first established in Edinburgh in 1996. These centers provide free practical, emotional, and social support to patients with cancer and their friends and family members. There are currently 20 such centers in the United Kingdom (mainly in National Health Service cancer hospitals) and other countries, and an online center has been established. Because the centers are places that anyone can casually visit at any time, it is difficult to maintain records for each individual and measure the effectiveness of the centers. However, the results of this study indicate that although it is difficult to increase health literacy, an individual resource, improving social support may lead to increased QOL in patients with cancer. This reinforces the importance of efforts to improve and facilitate social support.

### Quality of Life, Health Literacy, and Social Support According to Age

Studies in older individuals have shown that QOL increases with health literacy and social support. This study showed similar positive associations between QOL and health literacy and social support in patients with cancer 50 years or older. Moreover, the association was stronger for social support than for health literacy. A negative association was observed with the interaction of health literacy and social support, but the coefficient was much lower than for health literacy and social support. Patients high in health literacy collect information actively and independently and come to conclusions based on that information. The information and support provided by people around the patient may be at odds with those conclusions. In that case, the patient may become confused by the discrepancy and not amenable to social support. A simple slope analysis of the interaction between health literacy and social support showed social support to be significantly associated with QOL regardless of the level of health literacy. However, the coefficient for social support was larger when health literacy was low than when it was high. This result differs from the finding that, in older individuals, social support was more positively associated with health in those high in health literacy [40]. This may be related to the fact that the participants in this study were patients with cancer. The finding that social support was more positively associated with QOL in patients with cancer 50 years or older with low health literacy may be explained as follows: increasing the social support of patients with cancer that have low health literacy can mitigate the negative effect of low health literacy on QOL, as was indicated in the 2004 report by Lee [33].

The results for patients with cancer younger than 50 years differed from patients with cancer 50 years or older. Studies in young patients with cancer have included those of the adolescents and young adults (AYA) generation. The QOL in the AYA generation patients with cancer has been found to be low [53-57]. Moreover, the financial, mental health, and support group services available for the AYA generation patients are inadequate [58-60], as these patients desire information on side effects, alternative treatment options, pregnancy and childbirth options, and long-term care [59,61,62]. The AYA generation patients have also been shown to fear a continual fight against cancer and to experience negative emotions related to financial problems, death, body image, and perceived stigmas [62,63]. Furthermore, the types of treatment, lack of insurance, and withdrawing from school or a job after diagnosis have negative effects on occupational and educational outcomes [63,64].

Relationships with friends, family members, and other cancer survivors have been shown to lead to improved QOL in the AYA generation patients [60,62]. However, siblings of these patients have been reported to experience high levels of psychological distress [65], and problems such as persistent negative emotions related to the diagnosis and stigma associated with cancer have been reported for the parents and caregivers of such patients [66]. Thus, it may be difficult for patients with cancer to receive adequate support from family members, who are the patients’ closest supporters.

Similar to the AYA generation, patients younger than 50 years are affected by cancer early and must therefore cope with the disease for a long time, making the mental burden greater than that of patients aged 50 years or older. Because there are generally few patients with cancer in the same age group, it will be important to examine further ways to strengthen the social support system based on their needs.

### Limitations

This retrospective study was conducted using an online survey. Consequently, a limitation of the study was that it was based on patient perceptions. Further investigations that consider factors such as the type and stage of cancer and the time since diagnosis are needed for young patients. Accordingly, it will be important to examine how to strengthen the support systems available to patients with cancer by elucidating their needs based on their current status.

### Conclusions

The results of this study showed that the QOL in patients with cancer increased with health literacy, social support, and age. Moreover, the relationship with QOL was stronger for social support than for health literacy; similar results were obtained for patients with cancer 50 years or older. These findings suggest the importance of health literacy and social support, which has been noted previously, and indicate that the support of those around the patient has a greater effect on QOL than health literacy.

Different results were obtained for patients with cancer younger than 50 years. QOL in this group was lower than that in those 50 years or older. In view of the problems faced by younger patients with cancer, which have been identified in previous studies, elucidating the needs of these patients and further strengthening social support based on those needs may lead to improvements in QOL.

## Abbreviations

AYA: adolescents and young adults
FACT-G: Functional Assessment of Cancer Therapy-General
FACT-G7: Functional Assessment of Cancer Therapy-General (7-item version)
HLS-EU-Q47: European Health Literacy Survey Questionnaire
QOL: quality of life
